# Disordering of Human Telomeric G-Quadruplex with Novel Antiproliferative Anthrathiophenedione

**DOI:** 10.1371/journal.pone.0027151

**Published:** 2011-11-15

**Authors:** Dmitry Kaluzhny, Nikolay Ilyinsky, Andrei Shchekotikhin, Yuri Sinkevich, Philipp O. Tsvetkov, Vladimir Tsvetkov, Alexander Veselovsky, Mikhail Livshits, Olga Borisova, Alexander Shtil, Anna Shchyolkina

**Affiliations:** 1 Engelhardt Institute of Molecular Biology, Russian Academy of Sciences, Moscow, Russian Federation; 2 Moscow Institute of Physics and Technology, Dolgoprudny, Russian Federation; 3 Gause Institute of New Antibiotics, Russian Academy of Medical Sciences, Moscow, Russian Federation; 4 Mendeleyev University of Chemical Technology, Moscow, Russia; 5 Orekhovich Institute of Biomedical Chemistry, Russian Academy of Medical Sciences, Moscow, Russian Federation; 6 Blokhin Cancer Center, Russian Academy of Medical Sciences, Moscow, Russian Federation; Roswell Park Cancer Institute, United States of America

## Abstract

Linear heteroareneanthracenediones have been shown to interfere with DNA functions, thereby causing death of human tumor cells and their drug resistant counterparts. Here we report the interaction of our novel antiproliferative agent 4,11-bis[(2-{[acetimido]amino}ethyl)amino]anthra[2,3-*b*]thiophene-5,10-dione with telomeric DNA structures studied by isothermal titration calorimetry, circular dichroism and UV absorption spectroscopy. New compound demonstrated a high affinity (K_ass_∼10^6^ M^−1^) for human telomeric antiparallel quadruplex d(TTAGGG)_4_ and duplex d(TTAGGG)_4_∶d(CCCTAA)_4_. Importantly, a ∼100-fold higher affinity was determined for the ligand binding to an unordered oligonucleotide d(TTAGGG TTAG**A**G TTAGGG TTAGGG unable to form quadruplex structures. Moreover, in the presence of Na^+^ the compound caused dramatic conformational perturbation of the telomeric G-quadruplex, namely, almost complete disordering of G-quartets. Disorganization of a portion of G-quartets in the presence of K^+^ was also detected. Molecular dynamics simulations were performed to illustrate how the binding of one molecule of the ligand might disrupt the G-quartet adjacent to the diagonal loop of telomeric G-quadruplex. Our results provide evidence for a non-trivial mode of alteration of G-quadruplex structure by tentative antiproliferative drugs.

## Introduction

The anthraquinone-containing antibiotics and their analogues are among the most efficacious antitumor drugs (see [1]–[3] for recent reviews). The formation of drug-DNA complexes is the established mechanism of cytotoxicity of anthraquinone derivatives [4]–[8]. The binding of small molecules to different DNA structures results in DNA damage and interference with DNA dependent enzymes, eventually leading to cell death. A series of 1,4- and 2,6-difunctionalized amidoanthracene-9,10-diones have been shown to inhibit human telomerase via stabilization of telomeric G-quadruplex structures [9], [10]. Guanine quadruplexes (G-quadruplexes) are formed via an association of four guanines into a cyclic Hoogsteen hydrogen bonding arrangement in which each guanine shares two hydrogen bonds with its neighbor (N1–O6 and N2–N7) [11]. The G-quartets formed by single strand telomeric sequence may regulate telomerase activity: the intramolecular G-quadruplexes block telomerase activity in vitro [12], [13]. The derivatives of anthracene-9,10-diones substituted at the positions 1,5, 1,8 and 2,7 formed 1∶1 end-capping complexes with human telomeric G-quadruplex and also effectively inhibited telomerase [14].

**Figure 1:**
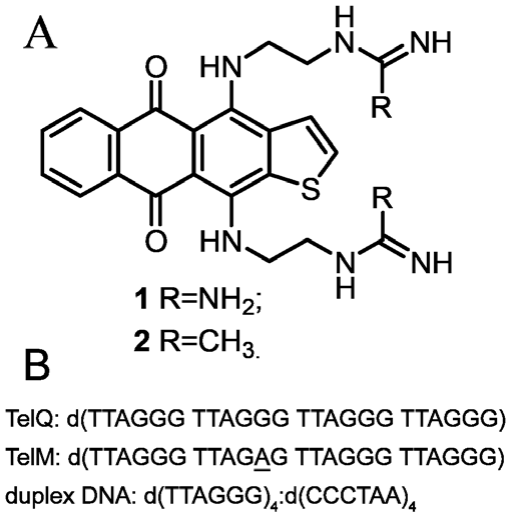
Structures of anthra[2,3-*b*]thiophene-5,10-diones 1 and 2 (A); oligonucleotides used in the study (B).

The emergence of drug resistance as well as organ toxicity may hamper the clinical efficacy of anthraquinone-based drugs [15]–[17]. Aiming at agents with improved chemotherapeutic properties, a series of linear heteroareneanthracenediones has been synthesized [18]. Some compounds of this chemotype showed a marked antiproliferative efficacy, in particular, a remarkable potency against cell lines with the determinants of drug resistance such as P-glycoprotein or p53 dysfunction. Besides, within the series of anthra[2,3-*b*]thiophene-5,10-dione we identified bis(guanidine) derivative (compound **1**; Fig. 1A) as a potent inhibitor of polydeoxynucleotide synthesis in TRAP assay [19]. One may suggest that strong basicity and delocalization of charges in the guanidine groups of side chains attributed to this effect.

However, it was difficult to determine the affinity of **1** to DNA structures due to poor water solubility and aggregation of the compound at physiological temperature, pH and ionic strength of buffers. To avoid these obstacles we prepared a novel analogue of **1** in which both guanidine groups in the side chains were substituted for acetamidines. This modification retained the basicity and the characteristic delocalization of terminal cationic centers in the side chains and improved the physical properties of the ligand. In the present study we report the binding of this novel anthrathiophenedione derivative, 4,11-bis[(2-{[acetimido]amino}ethyl)amino]anthra[2,3-*b*]thiophene-5,10-dione (compound **2**; Fig. 1A) to telomeric DNA duplex, telomeric G-quadruplex d(TTAGGG)_4_ (TelQ) and the mutant G11A oligonucleotide d(TTAGGG TTAGAG TTAGGG TTAGGG) (TelM) (Fig. 1B) unable to form quadruplex structures in the presence of Na^+^ [20]. Two conformations of intramolecular TelQ were studied∶ antiparallel G-quadruplex TelQ_Na_ formed in the presence of Na^+^ and a ‘3+1’ hybrid G-quadruplex conformation (TelQ_K_) that can be stabilized by K^+^ counterions [21]. We found a high affinity of **2** to DNA structures, with a ∼100-fold preference for G-containing unordered DNA in the presence of Na^+^. Moreover, the addition of **2** to the antiparallel G-quadruplex in the presence of Na^+^ led to circular dichroism (CD) and UV absorption spectral changes compatible with nearly complete unfolding of the antiparallel quadruplex conformation. Partial unfolding of TelQ_K_ by **2** occurred in the presence of K^+^. Molecular dynamics simulations illustrated a mode of TelQ_Na_ disordering by **2**.

## Results and Discussion

### Synthesis and cytotoxicity of compound 2

Similarly to the previously reported compound **1** [19], the synthesis of **2** was performed by modification of side chains in 4,11-bis[(2-aminoethyl)amino]anthra[2,3-*b*]thiophene-5,10-dione (compound **3** in Scheme S1) by amidation of terminal amino groups with ethyl acetimidate hydrochloride (Scheme S1; see Section S1 for details). After this modification of the side chains of **1** the basicity and the characteristic delocalization of terminal cationic centers in **2** remained unaltered. However, this modification diminished the polarity, increased the water solubility and decreased the intermolecular association of molecules of **2** in aqueous solutions. Testing of antiproliferative potency revealed that **2** inhibited the viability of leukemia (L1210, Molt4/C8, CEM, K562) and colon carcinoma HCT116 cell lines at micromolar concentrations after 72 h of exposure (IC_50_, a concentration that inhibited cell viability by 50%, was 5–10 µM as determined by MTT-test [19]. Importantly, **2** was similarly potent against the sublines otherwise resistant to structurally related antitumor DNA intercalator doxorubicin due to expression of the transmembrane transporter P-glycoprotein or deletion of p53 [19]. These data suggested that the modification of the side chain in **1** yielded the compound with promising anticancer characteristics. We set out to investigate the interaction of **2** with various DNA structures.

### Thermodynamic parameters of DNA:2 complex formation

The interactions of **2** with duplex DNA (Fig. 1B) as well as with quadruplex–forming oligonucleotide TelQ and the oligonucleotide TelM (unable to fold in an intramolecular quadruplex) were studied with isothermal titration calorimetry (ITC) in the presence of NaCl or KCl (Fig. S1). The thermodynamic parameters are presented in Table 1. Compound **2** binds to double stranded DNA with ***K*** **_ass_** = 2.2±0.1·10^6^ M^−1^, with the stoichiometry N_1_ = 5–6 molecules per 24 bp telomeric duplex. Up to three molecules of **2** can bind to the antiparallel G-quadruplex d(TTAGGG)_4_ in the presence of NaCl (TelQ_Na_) with *K*_a**ss**_∼10^6^ M^−1^. In the presence of NaCl the unordered oligonucleotide TelM binds **2** much stronger (*K* **_ass_** = 2.0±0.7·10^8^ M^−1^ for one molecule and *K*_a**ss**_∼5.7·10^6^ M^−1^ for the next six molecules; Table 1). KCl ensures a tight ligand binding to TelQ_K_ (*K* **_ass_** = 1.0±0.15·10^7^ M^−1^) with the maximal number of binding sites N = 7. The thermodynamic profiles of binding of **2** to dsDNA, TelM and TelQ (Table 1) in most cases corresponded to the induced-fit mechanism. A large favorable enthalpy contribution and a rather modest, though significant, unfavorable entropic contribution to the binding free energy are characteristic for the coupling of ligand binding to a major conformational change in DNA creating a stable binding pocket [22]. The entropic gain at the binding of **2** to dsDNA should be due to the effect of hydrophobic transfer. The CD spectra of dsDNA indicate a decreased base stacking interaction upon the ligand binding (Fig. S2). We anticipate that **2** semi-intercalates into dsDNA while the bulky groups are located in the duplex groove(s). The difference between TelQ_Na_ and TelQ_K_ in the strength of binding to **2** is due to the difference in the losses of entropy associated with various degrees of conformational changes of the quadruplexes. TelQ_K_ is likely to undergo incomplete conformational change. Thus, two modes of binding of **2** to TelM were revealed, suggesting that the one site of stronger, enthalpy driven binding is the intercalation site whereas about six weaker ones with nearly null entropic component correspond to external binding.

**Table 1:** Thermodynamic parameters of binding of 2 to oligonucleotides.

	ΔH, kcal/mol	−TΔS, kcal/mol	*K*_ass_×10^−6^, M^−1^	N
TelQ, NaCl	−12.1±0.4	3.9	0.9±0.1	3.0±0.1
TelQ, KCl	−12.0±0.1	2.4	10.6±1.7	7.0±0.1
TelM, NaCl	−16.2±0.6	4.9	200±70	1.0
	−9.4±0.2	0.2	5.7±0.7	5.8
dsDNA	−5.3±0.1	−3.4	2.2±0.1	5.6±0.1

ITC was performed in 10 mM Na phosphate buffer, pH 6.5 supplemented with 100 mM NaCl or 100 mM KCl, *T* = 25°C. K_ass_ is the association constant, ΔH is enthalpy changes, and N is the maximal number of bound molecules of **2** per DNA. The entropy variation (ΔS) was calculated according to the standard thermodynamic equation.

### Drug-TelQ_Na_ interaction monitored by circular dichroism

**Figure 2:**
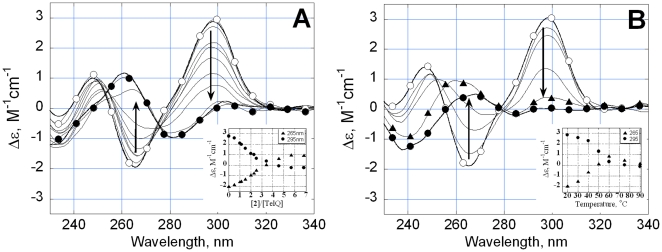
CD spectra of TelQ. (A) CD spectra of TelQ at various concentrations of 2. INSET: CD magnitudes at 295 nm (filled circles) and 265 nm (triangles). The concentration of TelQ oligonucleotide was 0.8 µM, the ratios of concentrations compound 2 to TelQ are given on the X axis (INSET). T = 20°C. (B) CD spectra of TelQ at 20°C (open circles), 30°C, 40°C, 50°C, 60°C (triangles), 75°C (filled circles). Samples contained 100 mM NaCl, 10 mM Na phosphate buffer, pH 7.6.

The CD spectrum of TelQ_Na_ is shown in Fig. 2A, open circles. The CD curve in the presence of Na^+^ reflects the folding of the oligonucleotide into an antiparallel G-quadruplex with two edgewise loops and a diagonal loop [21], [23]. The addition of **2** up to 6 µM dramatically changed the CD spectra (Fig. 2A, filled circles). Importantly, upon the binding of the ligand to the quadruplex structure no CD induction in the region of absorption of **2** (450–700 nm) was detectable. We assume that the ligand-associated CD changes below 320 nm reflected DNA conformational changes rather than CD induction. The magnitudes of the positive CD band centered at 295 nm and the negative band at 265 nm decreased (Fig. 2A). We next compared the CD spectrum of TelQ_Na_:**2** complex (Fig. 2A, filled circles) with CD spectra of TelQ_Na_ denatured by high temperature. Strikingly, the CD spectrum of the markedly denatured G-quadruplex at 60°C (Fig. 2B, triangles) was similar to the CD of TelQ_Na_:**2** complex (Fig. 2A, filled circles). The unfolding was almost complete after the addition of three ligand molecules per TelQ_Na_ (Fig. 2A, INSET), in agreement with the maximal number of bound ligand molecules determined by ITC (Table 1). Thus, the CD data suggested that the binding of **2** to the antiparallel TelQ_Na_ is accompanied by a significant unfolding of the TelQ_Na_. This suggestion is strengthened by data on thermal denaturation of TelQ_Na_ and TelQ_Na_:**2** monitored by absorption at λ = 295 nm (see below).

### Disruption of TelQ_Na_ structure by compound 2

**Figure 3:**
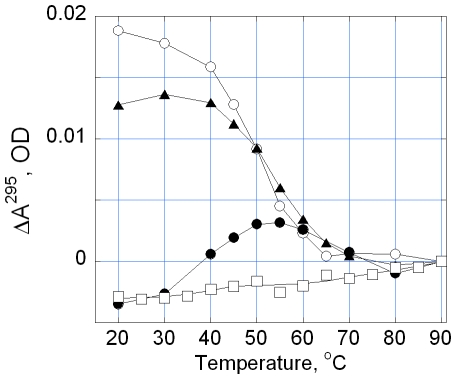
Thermal denaturation profiles of TelQ_Na_ and TelM. The temperature induced changes in absorption at 295 nm of TelQ_Na_ and TelM at difenent concentrations of compound **2**. Concentrations: TelQ was 1 µM, compound **2**: 0 (open circles), 1 µM (triangles) and 6 µM (filled circles). Samples contained 100 mM NaCl, 10 mM Na phosphate buffer, pH 7.6. The absorption of compound **2** was subtracted from the absorption of the sample at 295 nm. TelQ, open circles; TelM, open squares.

The UV melting of TelQ_Na_ in the presence of **2** monitored by absorption at λ = 295 nm is shown in Fig. 3. This wavelength is particularly suitable for detection of quadruplexes because absorption at λ = 295 nm reflects the presence of G-quartets [24]. The melting profile for TelM (open squares) demonstrated the absence of a G-quartet structure in the range of temperatures 20°–90°C [20]. The melting experiment shows a decreased absorption at 295 nm at 20°C upon the addition of 1 µM of **2** to TelQ_Na_, indicating a partial loss of G-quartet structure (Fig. 3, triangles). After the addition of 6 µM of **2** the quadruplex conformation of TelQ_Na_ was abolished at 20°C (Fig. 3, filled circles). Upon heating up to 55–60°C, the melting curve for TelQ_Na_:**2** complex became indistinguishable from that for free (no ligand) TelQ_Na_ (Fig. 3, open circles). Apparently, **2** dissociates from the oligonucleotide during heating; the complete DNA clearance occurs at >55–60°C. As a result, a fraction of G-quadruplexes is restored at these temperatures followed by complete quadruplex denaturation at >80–90°C. Thus, the absorption data independently confirmed the loss of G-quartets in TelQ_Na_:**2** complexes in the presence of Na^+^. We emphasize that the term ‘disordering’ of the studied TelQ_Na_ implies complete loss of G-quartets. Furthermore, interactions between all bases in TelQ_Na_ structure significantly decreased upon binding of **2** as revealed with CD (Fig. 2A). The conformation of the sugar-phosphate backbone in the ‘disordered’ state remains to be determined.

Comparison of TelQ_Na_:**2** and TelM:**2** complexes demonstrated differential affinity of the ligand to these oligonucleotides; the maximal number of bound molecules of **2** also differed (Table 1). In contrast to TelQ_Na_, the conformation of free TelM oligonucleotide under the conditions of our experiments (DNA concentrations, ionic strength of buffers, room temperature) was represented predominantly by a single DNA strand with a few hydrogen bonds between nucleotides (Fig. S3). The addition of **2** led to the formation and/or stabilization of a conformation, most likely of an imperfect TelM hairpin, with a high affinity intercalation site for **2** (*K*_ass_≈2·10^8^ M^−1^) (Table 1). The saturated TelM:**2** complex presumably includes a TelM hairpin with two tightly intercalated and five externally bound drug molecules probably located at the stem of the TelM hairpin. This conclusion is consistent with ITC data (see section *Thermodynamic parameters of DNA:* ***2*** *complex formation*). This consideration allows us to suggest that the effect of **2** on the single stranded telomeric DNA may directly depend on the propensity of the nucleotide sequence to form a quadruplex or a hairpin. In the former case, the DNA bound to **2** is disordered whereas in the latter scenario, the DNA stretch folds into a hairpin stabilized by the intercalating ligand. In other words, **2** can evoke a dual effect on DNA structure, that is, to break the quadruplex and to stabilize the hairpins.

### Interaction of compound 2 with TelQ_K_

**Figure 4:**
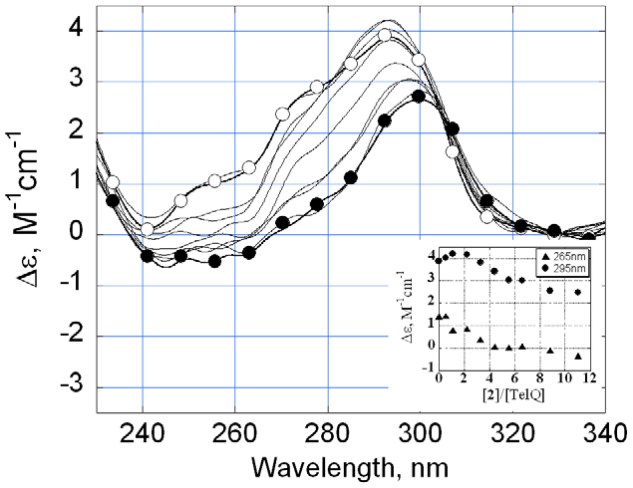
Circular dichroism spectra of TelQ at various concentrations of compound 2 in the presence of 0.1 M KCl. INSET: CD magnitudes at 295 nm (filled circles) and 265 nm (triangles). The concentration of TelQ oligonucleotide was 1 µM, the ratios of concentrations compound **2** to TelQ are given on the X axis (INSET). *T* = 20°C. Samples contained 100 mM KCl, 10 mM Na phosphate buffer, pH 7.6.

In the presence of KCl the affinity of **2** to TelQ_K_ (*K*_ass_∼10^7^ M^−1^) and the maximal number of bound molecules of **2** (N = 7) were much greater than the respective values for TelQ_Na_ (Table 1). The CD spectrum in 100 mM KCl revealed a ‘3+1’ folding of TelQ_K_ (Fig. 4, open circles) [25]. Three ‘strands’ of this intramolecular fold run parallel to one another, while the fourth ‘strand’ is antiparallel [26]. Theoretical calculations of the CD spectrum [25] identified the stacking mode of three G-quartets, two of which stack with the same polarity whereas the third one stacks with different polarity, independently proving the mode of quadruplex folding suggested by NMR [26]. The addition of **2** to TelQ_K_ resulted in another shape of CD signal of the quadruplex (Fig. 4, filled circles). This spectrum is compatible with an antiparallel G-quadruplex, where the quartets stack with different polarities [25]. UV melting experiments shed light on G-quadruplex conformational changes upon ligand binding (see below).

### Compound 2 causes partial disorganization of TelQ_K_

**Figure 5:**
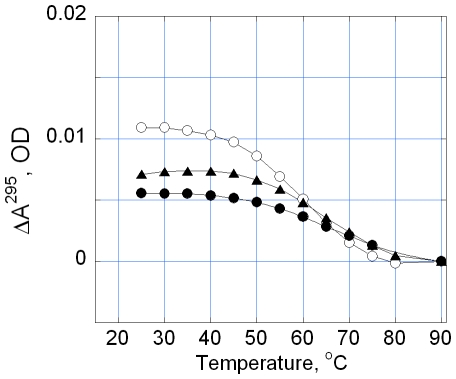
Thermal denaturation profiles of TelQ_K_ in the presence compound 2. Concentrations: TelQ 1 µM; compound **2**: 0 (open circles), 1 µM (triangles) and 2 µM (filled circles). Samples contained 0.1 M KCl, 10 mM Na phosphate buffer, pH 7.6. The contribution of compound **2** to the absorption of the sample at 295 nm was subtracted.

We monitored the temperature dependence of TelQ_K_ absorption at 295 nm at different concentrations of **2** in the presence of 100 mM KCl (Fig. 5). At equal or close molar ratios TelQ_K_:**2** the absorption at 295 nm decreased, which normally indicates partial loss of G-quartets (Fig. 5, triangles and circles, respectively). Further increase of concentration of **2** led to opalescence of the sample that prevented reliable measurements. Probably, certain intermolecular interactions or drug aggregation at higher concentrations occurred in the presence of KCl, thereby causing light scattering. Thus, the absorption method provided evidence that, in the presence of **2** and KCl, at least a portion of the G-quartets in TelQ_K_ became disordered. The CD spectrum of the saturated complex TelQ_K_:**2** (Fig. 4, filled circles) is consistent with the presence of two quartets with different polarities [25], whereas the third G-quartet of the TelQ_K_ oligonucleotide may be broken upon ligand binding. The loss of the quartet that stacked with the same polarity as the neighboring quartet may account for the observed decrease of the CD signal around 260–265 nm (Fig. 4, INSET, triangles).

Partial disordering of TelQ_K_ conformation by **2** was further substantiated by the thermodynamic parameters of TelQ_K_:**2** complex (see *Thermodynamic parameters of DNA:* ***2*** *complex formation*). The loss of the G-quartet structure should result in lengthening of the loops that connect two remaining G-quartets. One loop may acquire single nucleotide (guanine) whereas two additional guanines would be added to another loop (see the structure in [26]). The single strand loops might be good candidates for binding to **2** (Table 1). Stabilization of the antiparallel ‘3+1’ TelQ_K_ in the presence of K^+^ counterions [27] is likely to preclude drug-induced disruption of the quadruplex conformation.

### Computer modeling of binding of 2 to TelQ_Na_

**Figure 6:**
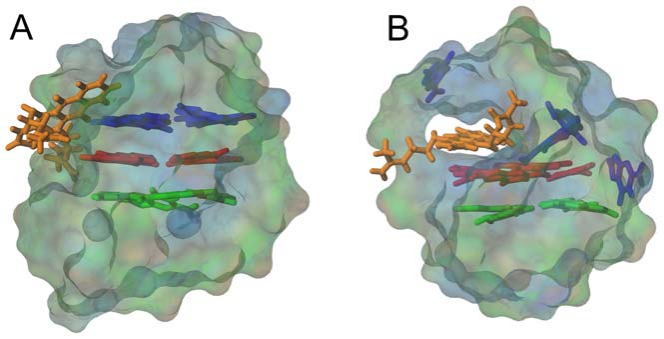
A model of the complex of compound 2 with antiparallel TelQ. (A) Docking of **2** to TelQ_Na_. (B) The snapshot after the initial 7 nsec of molecular dynamics simulations. Compound **2** is rendered in light brown color; blue, red and green are the guanines of TelQ. The water accessible surface is shown. Colors are given according to an increase in log P values from brown to blue.

Fig. 6A shows the results of docking of **2** to the rigid structure of TelQ_Na_ ([28], PDB ID: 143D). The position of the ligand with the lowest energy was selected for molecular dynamics simulations. The results obtained after 7 nsec of simulation of the complex TelQ_Na_:**2** demonstrated that even the binding of one molecule of **2** disrupted the G-quartet adjacent to the diagonal loop (Fig. 6B). Despite the loss of this G-quartet the sugar-phosphate backbone remained unaltered. The positioning of a highly hydrophobic tetracyclic backbone in **2** is the most favorable inside the cavity of the quadruplex. This fact, as well as limited space for capping due to the size and geometry of **2**, might explain the disruption of the G-quartet by this ligand. The side chains in **2** provide the orientation of the ligand inside the cavity and displacement of guanines from the G-quartet plane. These simulations are in good agreement with our experimental data that strongly suggested that **2** forms high affinity complexes with the telomeric G-quadruplex, and this process involves disruption of the G-quartets.

In summary, the novel compound 4,11-bis[(2-{[acetimido]amino}ethyl)amino]anthra[2,3-*b*]thiophene-5,10-dione (**2**) demonstrated a high affinity (*K*_ass_≈10^6^–10^8^ M ^−1^) to double helical DNA, TelQ and an unordered TelM oligonucleotide. The binding of the ligand to these DNA targets may exert biological effects on cell functions and survival. Intercalation into dsDNA may lead to inhibition of template synthesis, thereby impeding gene expression and function of DNA dependent enzymes. These effects are common for many analogues of anthracene-9,10-diones [19]. Apart from the intercalation into dsDNA, we found a preference of **2** to unordered DNA conformation. Compound **2** disorders the intramolecular quadruplex conformation rather than stabilizing the quadruplex. Thus, **2** may exert a remarkable effect on G-containing strands present in the promoter regions of oncogenes. These sequences are involved in gene regulation via G-quadruplex formation, impeding recruitment of proteins necessary for gene expression [29], [30]. The antiproliferative activity of **2** may also be associated with tight binding to the G-forming sequences and competing with binding of regulatory proteins to DNA. In vitro, the d(TTAGGG)_4_ sequence is able to fold into two major G-quadruplex forms depending on the type of a counterion and designated in the study as TelQ_Na_ and TelQ_K_. The DNA environment in the nucleus also includes proteins, low molecular substances and a significant local dehydration. Which G-quadruplex form, TelQ_Na_ or TelQ_K_, of the telomeric sequence is realized in vivo, if any, has not been proven so far. Design of telomeric DNA ligands aimed at stabilization of G-quadruplex has been reported in the literature [29], [30], and some G-quadruplex stabilizers proved to be efficient antiproliferative agents. Our data suggest a non-trivial explanation of antiproliferative efficacy of the drug, that is, a competition of the ligand with cellular proteins for binding to the telomeric ssDNA rather than G-quadruplex stabilization. One can suppose that the ability to deorganize DNA quadruplexes may be potentially valuable anticancer property of anthrathiophenedione derivatives.

## Materials and Methods

### Reagents

The oligonucleotides d(TTAGGG)_4_ (TelQ), d(TTAGGGTTAGAG(TTAGGG)_2_) (TelM) and d(CCCTAA)_4_:d(TTAGGG)_4_ (ds DNA) were synthesized by Syntol (Moscow). The DNA preparations were dissolved in the buffers containing 10 mM sodium phosphate supplemented with 100 mM NaCl or 100 mM KCl, pH 6.5 or 7.6. 4,11-Bis[(2-{[acetimido]amino}ethyl)amino]anthra[2,3-b]thiophene-5,10-dione dihydrochloride (compound **2**) was dissolved in dimethylsulfoxide as 10 mM stock solution and kept at 4°C. Aqueous solutions of **2** were prepared immediately before the experiments.

### Spectral methods

UV absorption spectra were acquired with a Jasco V-550 spectrophotometer (Japan). CD spectra were recorded on a Jasco 715 spectropolarimeter (Japan). The instruments were equipped with Peltier thermostated cell holders. Molar dichroism (Δε) was calculated per mole of nucleotides.

### Isothermal titration calorimetry

The thermodynamic parameters of binding of **2** to oligonucleotides were measured using an iTC200 instrument (MicroCal, Northampton, MA). Experiments were carried out at 25°C in 100 mM NaCl or 100 mM KCl, 10 mM sodium phosphate buffer, pH 6.5. Two microliter aliquots of solution of **2** were injected into a 200 µl calorimetric cuvette containing the oligonucleotide solution to achieve the complete binding isotherm. The concentrations of oligonucleotides in the cell were 1.5–5 µM; the concentration of **2** in the syringe ranged from 100 to 200 µM. The heat of dilution was measured by injecting the solution of **2** into the buffer. The obtained values were subtracted from the heat of reaction to obtain the effective heat of binding. The resulting titration curves were fitted using ‘two set of sites’ model on MicroCal Origin software. Thus, the association constant K_ass_, enthalpy changes ΔH and stoichiometry N were determined. The entropy variation (ΔS) was calculated according to the standard thermodynamic equation.

### Molecular modeling

The coordinates of the atomic positions of TelQ were taken from NMR (PDB ID: 143D). The model of compound **2** was created using SYBYL 8.0 molecular modeling package (Tripos Inc., St. Louis, USA). To define the most probable binding site for **2** in TelQ, the procedure of flexible ligand docking to the full surface of a rigid G-quadruplex was performed using DOCK 6.4 and the Anchor-and-Grow algorithm. The best docking pose was selected based on secondary scoring function of DOCK 6.4. The follow-up analysis of the ligand's influence on G-quadruplexes was performed using the molecular dynamics (MD) simulation with the application of a suite of programs Amber 8 in implicit solvent using general Born model. The MD simulations in the production phase were carried out using constant pressure on a trajectory of 10 ns with 2 fs step. For more details of molecular docking and MD simulation see Section S2.

## Supporting Information

Section S1 Synthesis of 4,11-bis[(2-{[acetimido]amino}ethyl)amino]anthra[2,3-b]thiophene-5,10-dione dihydrochloride (compound **2**).
(PDF)

Scheme S1 Synthesis of 4,11-bis[(2-{[acetimido]amino}ethyl)amino]anthra[2,3-*b*]thiophene-5,10-dione (**2**).
(PDF)

Section S2 Molecular modeling.
(PDF)

Figure S1 Isothermal titration calorimetry of DNA:**2** complexes.
(PDF)

Figure S2 CD spectra of the telomeric duplex d(TTAGGG)4:d(CCCTAA)4 at various concentrations of **2**.
(PDF)

Figure S3 CD spectra of TelM:**2** complexes.
(PDF)
