# Supplementary material for: Disordering of Human Telomeric G-Quadruplex with Novel Antiproliferative Anthrathiophenedione
Source: PLoS One. 2011 Nov 15;6(11):e27151. doi: 10.1371/journal.pone.0027151 (PMC3216923; doi:10.1371/journal.pone.0027151)
Supplement: Section S1 — Synthesis of 4,11-bis[(2-{[acetimido]amino}ethyl)amino]anthra[2,3-b]thiophene-5,10-dione dihydrochloride (compound 2). (PDF) [file pone.0027151.s001.pdf]

Section S1

Synthesis of 4,11-bis[(2-{[acetimido]amino}ethyl)amino]anthra[2,3-b]thiophene-5,10-dione dihydrochloride (compound 2).

The starting anthra[2,3-*b*]thiophene-5,10-dione **3** [1] (100 mg, 0.26 mmol) was dissolved in warm dimethylsulfoxide (10.0 mL). Methanol (10 mL), triethylamine (0.16 mL, 1.1 mmol) and hydrochloride of ethyl acetimidate (0.2 g, 1.1 mmol) were subsequently added, the mixture was stirred for 3 h at 45ºC, then cooled and quenched with acetone. The blue solid precipitate was purified by reverse phase chromatography (H_2O–MeCN-HCO_2H, 50:10:1 v/v). The residue obtained after purification and evaporation was dissolved in warm MeOH, then the solution of 1N HCl in MeOH (0.5 mL) was added and the product was re-precipitated with acetone. The solid was collected, washed with acetone and dried. The yield of dihydrochloride **2** 53 mg (38 %); mp >250 ºC (dec.); HPLC Kromasil-100-5-mkm C-18 column (4.6×250 mm, LW=564 nm), eluent: A – H_3PO_4 (0.01M), B – MeCN; gradient B 15 → 40% (20 min), elution time 11.6 min, purity 95%. ^1H NMR δ: 8.24 (m, 2H, 6-H, 9-H), 7.98 (d, 1H, 3-H), 7.90 (d, 1H, 2-H), 7.71 (m, 2H, 7-H, 8-H), 4.15 (m, 2H, HNCH_2), 4.01 (m, 2H, HNCH_2), 3.64 (m, 4H, CH_2NH), 2.26 (s, 3H, Me), 2.24 (s, 3H, Me); UV (ethanol) λ_{max} (log ε) 235 sh (4.2), 267 (4.6), 285 sh (4.0), 325 sh (3.7), 394 (3.2), 522 sh (3.8), 563 (4.2), 605 (4.3) nm; HRMS (ESI) calculated for $C_{24}H_{26}N_6O_2S$: 462.1838, found $[M+H]^+$ 463.1843.

NMR spectra were registered on a Varian VXR-400 instrument operated at 400 MHz (^1H NMR). Chemical shifts were measured in CD_3OD using tetramethylsilane as internal standard. Column chromatography was performed on silanized Silica Gel Merck 60. Melting points were determined on a Buchi SMP-20 apparatus and are uncorrected. High resolution mass spectra were recorded with electron-spray ionization on a Bruker Daltonics microOTOF-QII instrument. UV spectra were recorded on Hitachi-U2000 spectrophotometer. HPLC was performed using Shimadzu Class-VP V6.12SP1 system. All solutions were evaporated at reduced pressure on a Buchi-R200 rotary evaporator at the temperature below 45 °C**.**

Supporting References.

1. Shchekotikhin AE, Glazunova VA, Dezhenkova LG, Luzikov YN, Sinkevich YB, et al. (2009) Synthesis and cytotoxic properties of 4,11-bis[(aminoethyl)amino]anthra[2,3-b]thiophene-5,10-diones, novel analogues of antitumor anthracene-9,10-diones. Bioorg Med Chem 17: 1861-1869.
