# Supplementary figures and images for: Disordering of Human Telomeric G-Quadruplex with Novel Antiproliferative Anthrathiophenedione
Source: PLoS One. 2011 Nov 15;6(11):e27151. doi: 10.1371/journal.pone.0027151 (PMC3216923; doi:10.1371/journal.pone.0027151)

### *Scheme S1.*

*Synthesis of 4,11-bis[(2-[[acetimido]amino]ethyl)amino]anthra[2,3-b]thiophene-5,10-dione (2).*

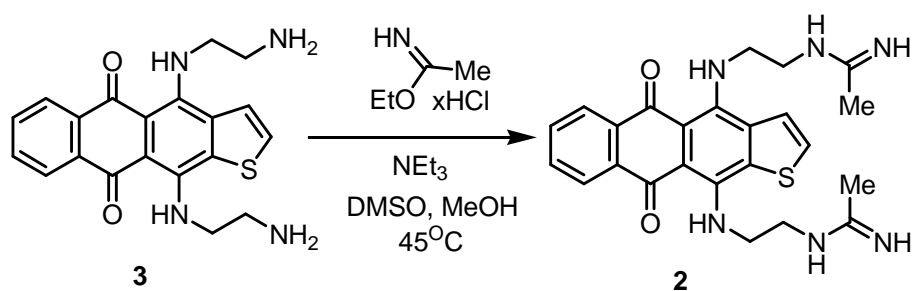

Supplement: Scheme S1 — Synthesis of 4,11-bis[(2-{[acetimido]amino}ethyl)amino]anthra[2,3-b]thiophene-5,10-dione (2). (PDF) [file pone.0027151.s002.pdf]
