# Supplementary material for: Disordering of Human Telomeric G-Quadruplex with Novel Antiproliferative Anthrathiophenedione
Source: PLoS One. 2011 Nov 15;6(11):e27151. doi: 10.1371/journal.pone.0027151 (PMC3216923; doi:10.1371/journal.pone.0027151)
Supplement: Section S2 — Molecular modeling. (PDF) [file pone.0027151.s003.pdf]

Section S2

Molecular modeling

The coordinates of the atomic positions of TelQ were taken from NMR ([1]; MMDB ID: 47630; PDB ID: 143D). The model of compound **2** was created using SYBYL 8.0 molecular modeling package (Tripos Inc., St. Louis, USA). Unfavorable van der Waals interactions in TelQ structure were removed by short minimizations using SYBYL 8.0 and Powell method [2] with the following parameters: TRIPOS force field [3], non-bond distance cutoff equal to 8 Å, gradient 0.05 kcal/mol·Å. For an initial optimization we used the simplex method with a cutoff set at 500 iterations with distance constraints between the donor H-bond and the acceptor atoms of guanines to maintain the conserved H-bond network of G-quartets. To calculate partial atomic charges for the ligand the quantum mechanical semi-empirical method PM3 [4] was used with the application of MOPAC 7.0 package included into Vega ZZ [5]. To determine partial atomic charges for TelQ atoms, the Gasteiger-Hückel method [6] was used.

To define the most probable binding site for **2** in TelQ, the procedure of flexible ligand docking to the full surface of a rigid G-quadruplex was performed using DOCK 6.4 and the Anchor-and-Grow algorithm. The solvent-accessible surface of the target for docking was built on the basis of the Connolly algorithm [7] with the probe radius 1.4 Å. Electrostatic and van der Waals potential fields generated over the target were performed prior to docking using the grid (spacing 0.3 Å). The non-bond distance cutoff was 12.0 Å, the parameters for van der Waals interactions were used from dw_AMBER_parm99.defn set. Compound **2** was docked using the grid-based energy scoring option for minimization after an initial placement in the site. Flexibility of **2** was modeled by regarding the compound as a series of fragments, where the central fragment (the anchor) was docked first followed by sequential docking of other fragments around the anchor. Once each fragment was docked, the neighboring fragments were combined [8]. The best docking pose was selected based on secondary scoring function of DOCK 6.4. This function used the solvatation implicitly through the generalized Born solvent-accessible surface area continuum model with salt screening [9]. The concentration of NaCl was 0.1 M.

The follow-up analysis of the ligand’s influence on G-quadruplexes was performed using the molecular dynamics (MD) simulation with the application of a suite of programs Amber 8 [10]. The complex obtained from docking was MD simulated in implicit solvent using general Born model [11] with the addition of 0.1 M NaCl. To calculate interatomic interaction energy the force fields GAFF for ligand and parmbsc0 [12] force field (a refinement of the AMBER parm99) for G-quadruplexes were used. The model was energy minimized using 250 steps of

steepest descent followed by 250 steps of conjugate gradient. Then gradual heating to 300 K during 20 ps was performed. To avoid wild fluctuations for our system at this stage, weak harmonic restrains were used with a force constant of 10 kcal* mol^{-1}*$Å^{-2}$ for all atoms of the complex except hydrogens. The SHAKE algorithm [13] was applied to constrain the bonds to hydrogen atoms, that allowed to use a 2 fs step. The MD simulations in the production phase were carried out using constant pressure on a trajectory of 10 ns with 2 fs step. To control the temperature Langevin thermostat was used with the collision frequency of 1 ps^{-1}.

Supporting References.

1. Wang Y, Patel DJ (1993) Solution structure of the human telomeric repeat d[AG3(T2AG3)3] G-tetraplex. Structure 1: 263-282.
2. Powell MJD (1977) Restart procedures for the conjugate gradient method. 241-254.
3. Clark M, Cramer RD, Van Opdenbosch N (1989) Validation of the general purpose tripos 5.2 force field. Journal of Computational Chemistry 10: 982-1012.
4. Stewart JJP (2007) Semiempirical Molecular Orbital Methods. Reviews in Computational Chemistry: John Wiley & Sons, Inc. pp. 45-81.
5. Pedretti A, Villa L, Vistoli G (2004) VEGA – An open platform to develop chemo-bio-informatics applications, using plug-in architecture and script programming. Journal of Computer-Aided Molecular Design 18: 167-173.
6. Gasteiger J, Marsili M (1978) A new model for calculating atomic charges in molecules. Tetrahedron Letters 19: 3181-3184.
7. Connolly ML (1983) Solvent-accessible surfaces of proteins and nucleic acids. Science 221: 709-713.
8. Zhou Z, Felts AK, Friesner RA, Levy RM (2007) Comparative Performance of Several Flexible Docking Programs and Scoring Functions: Enrichment Studies for a Diverse Set of Pharmaceutically Relevant Targets. Journal of Chemical Information and Modeling 47: 1599-1608.
9. Hawkins GD, Cramer CJ, Truhlar DG (1996) Parametrized Models of Aqueous Free Energies of Solvation Based on Pairwise Descreening of Solute Atomic Charges from a Dielectric Medium. The Journal of Physical Chemistry 100: 19824-19839.
10. Case DA, Darden TA, T.E. Cheatham I, C.L. Simmerling, J. Wang, et al. (2004) AMBER 8. University of California, San Francisco.
11. Onufriev A, Bashford D, Case DA (2004) Exploring protein native states and large-scale conformational changes with a modified generalized born model. Proteins 55: 383-394.
12. Perez A, Marchan I, Svozil D, Sponer J, Cheatham TE, 3rd, et al. (2007) Refinement of the AMBER force field for nucleic acids: improving the description of alpha/gamma conformers. Biophys J 92: 3817-3829.
13. Ryckaert J-P, Ciccotti G, Berendsen HJC (1977) Numerical integration of the cartesian equations of motion of a system with constraints: molecular dynamics of n-alkanes. Journal of Computational Physics 23: 327-341.
