# Supplementary material for: Disordering of Human Telomeric G-Quadruplex with Novel Antiproliferative Anthrathiophenedione
Source: PLoS One. 2011 Nov 15;6(11):e27151. doi: 10.1371/journal.pone.0027151 (PMC3216923; doi:10.1371/journal.pone.0027151)
Supplement: Figure S1 — Isothermal titration calorimetry of DNA:2 complexes. (PDF) [file pone.0027151.s004.pdf]

Figure S1.

Isothermal titration calorimetry of DNA:2 complexes.

(A) $TelQ_{Na}$; (B) $TelQ_K$; (C) TelM; (D) ds DNA.

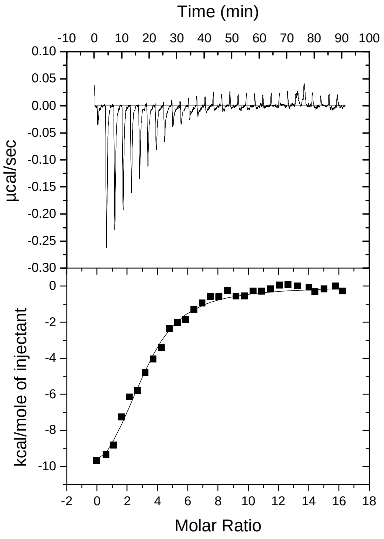

A

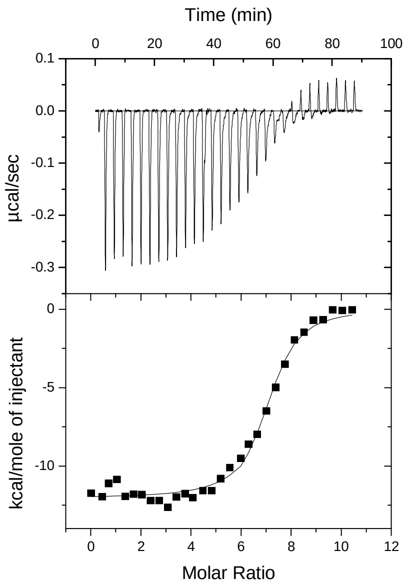

B

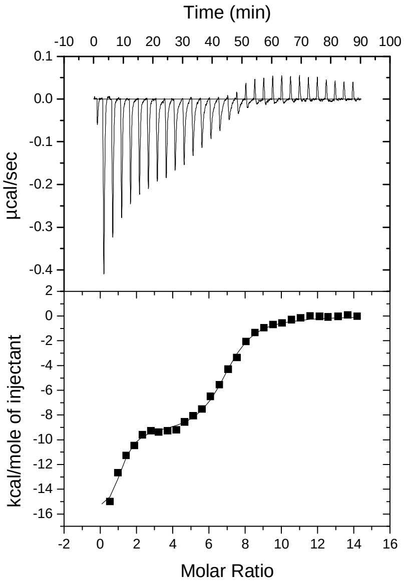

C

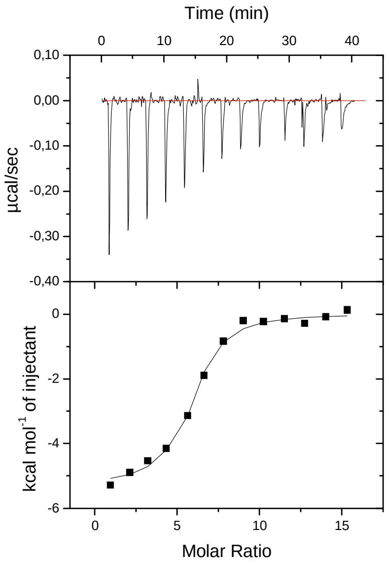

D
