# Supplementary material for: Disordering of Human Telomeric G-Quadruplex with Novel Antiproliferative Anthrathiophenedione
Source: PLoS One. 2011 Nov 15;6(11):e27151. doi: 10.1371/journal.pone.0027151 (PMC3216923; doi:10.1371/journal.pone.0027151)
Supplement: Figure S2 — CD spectra of the telomeric duplex d(TTAGGG)4:d(CCCTAA)4 at various concentrations of 2. (PDF) [file pone.0027151.s005.pdf]

Figure S2.

CD spectra of the telomeric duplex d(TTAGGG)$_4$:d(CCCTAA)$_4$ at various concentrations of 2.

The concentration of duplex was 0.5 µM, concentration of **2** varied from 0.05 µM to 3.2 µM. The samples contained 100 mM NaCl and 10 mM sodium phosphate buffer, pH 7.6 at 20°C.

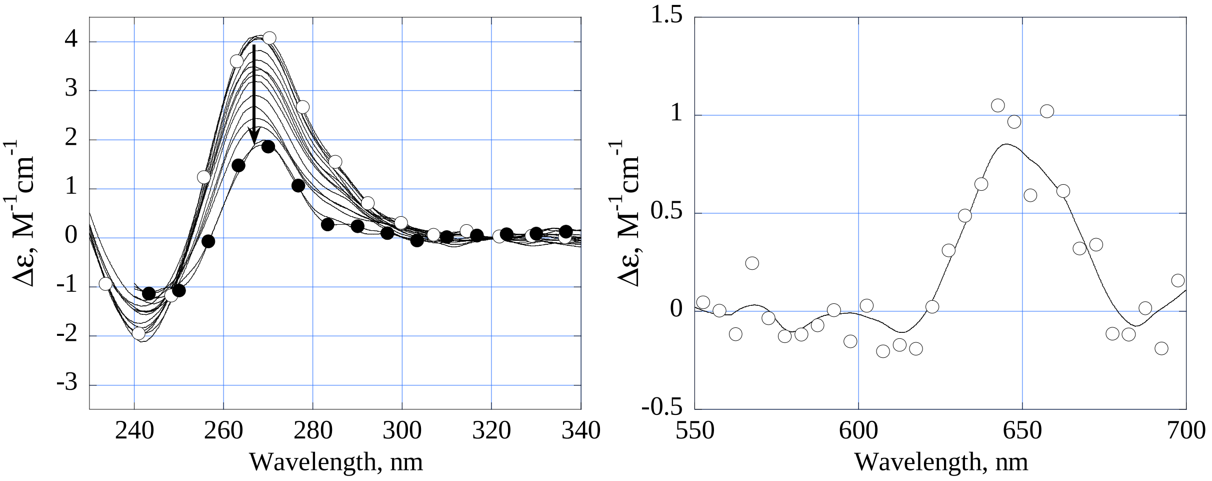

The CD spectra of dsDNA at various ligand concentrations are given in Figure S2. The shape of CD spectrum was conserved, whereas the magnitudes of the positive (270 nm) and negative (240 nm) bands decreased upon the addition of **2**. Also, an induced CD appeared in the region of the ligand absorption 600-680 nm [1].

Supporting References.

1. Kolodziejczyk P, Garnier-Suillerot A (1987) Circular dichroism study of the interaction of mitoxantrone, ametantrone and their Pd(II) complexes with deoxyribonucleic acid. Biochim Biophys Acta 926: 249-257.
