# Supplementary material for: Disordering of Human Telomeric G-Quadruplex with Novel Antiproliferative Anthrathiophenedione
Source: PLoS One. 2011 Nov 15;6(11):e27151. doi: 10.1371/journal.pone.0027151 (PMC3216923; doi:10.1371/journal.pone.0027151)
Supplement: Figure S3 — CD spectra of TelM:2 complexes. (PDF) [file pone.0027151.s006.pdf]

Figure S3.

CD spectra of TelM:2 complexes.

(A) dependence of CD signal on concentrations of **2**. (B) CD magnitudes at 295 nm (filled circles) and 265 nm (triangles). The samples contained 100 mM NaCl and 10 mM sodium phosphate buffer, pH 7.6 at 20°C. The concentration of TelM oligonucleotide was 1 μM, the ratios of concentrations TelM:**2** are given on the X axis(B).

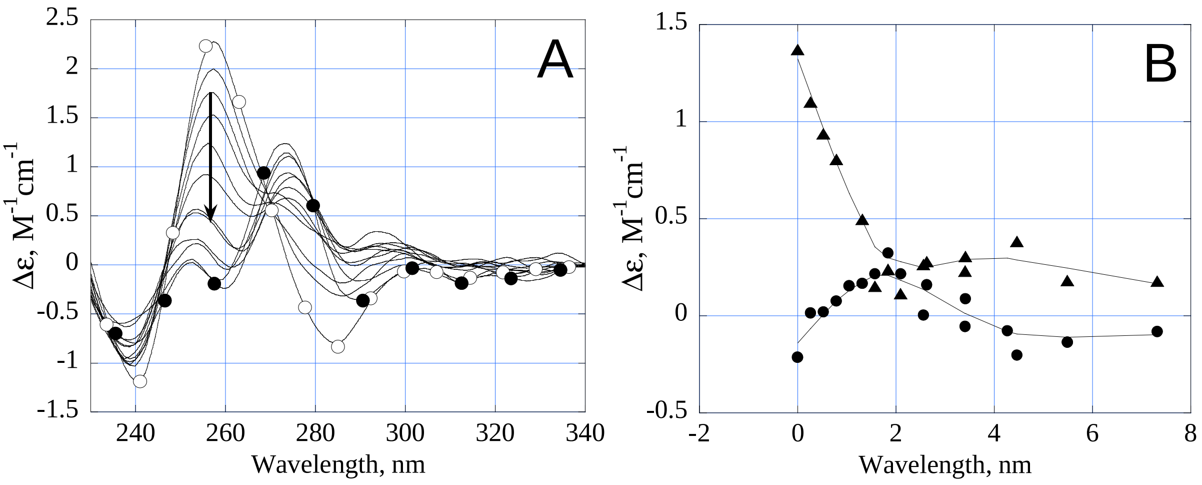

The CD spectrum of free TelM (open circles) is close to the sum of CD spectra of non-interacting mononucleotides comprising TelM sequence. This supports a single strand conformation of the oligonucleotide under given experimental conditions. The binding of two molecules of **2** changed the CD spectrum (filled circles), thereby reorganizing the oligonucleotide conformation. The binding of an additional 5 molecules of **2** detectable by ITC exerted little or no effect on the oligonucleotide conformation as determined by CD. The two proposed types of binding of **2** to TelM are discussed in the text.
